# Jasmonate Signalling and Defence Responses in the Model Legume *Medicago truncatula*—A Focus on Responses to *Fusarium* Wilt Disease

**DOI:** 10.3390/plants5010011

**Published:** 2016-02-05

**Authors:** Louise F. Thatcher, Ling-Ling Gao, Karam B. Singh

**Affiliations:** 1 CSIRO Agriculture, Centre for Environment and Life Sciences, Wembley, Western Australia 6913, Australia; Lingling.Gao@csiro.au (L-L.G.); Karam.Singh@csiro.au (K.B.S.); 2 The Institute of Agriculture, The University of Western Australia, 35 Stirling Highway, Crawley, Western Australia 6009, Australia

**Keywords:** methyl jasmonate, pathogen, necrotroph, *Fusarium*, Medicago, barrel medic, *Tnt*-insertion, gene expression, signaling

## Abstract

Jasmonate (JA)-mediated defences play important roles in host responses to pathogen attack, in particular to necrotrophic fungal pathogens that kill host cells in order to extract nutrients and live off the dead plant tissue. The root-infecting fungal pathogen *Fusarium oxysporum* initiates a necrotrophic growth phase towards the later stages of its lifecycle and is responsible for devastating *Fusarium* wilt disease on numerous legume crops worldwide. Here we describe the use of the model legume *Medicago truncatula* to study legume–*F. oxysporum* interactions and compare and contrast this against knowledge from other model pathosystems, in particular *Arabidopsis thaliana–F. oxysporum* interactions. We describe publically-available genomic, transcriptomic and genetic (mutant) resources developed in *M. truncatula* that enable dissection of host jasmonate responses and apply aspects of these herein during the *M. truncatula-–F. oxysporum* interaction. Our initial results suggest not all components of JA-responses observed in *M. truncatula* are shared with Arabidopsis in response to *F. oxysporum* infection.

## 1. Introduction

### 1.1. Pathogen Background

*Fusarium oxysporum* is a globally ubiquitous soil-borne fungus capable of infecting over 100 different plant species. This root-infecting fungus causes *Fusarium* wilt disease characterised by obstruction of the vascular system and the appearance of wilting. Initial root penetration is through wounds or at natural openings at the base of lateral root initials, followed by colonisation of the vascular system where spores, hyphal growth and the action of secreted pathogen toxins clog the xylem vessels and the flow of water. This is exacerbated by the action of host defences aiming to limit pathogen spread but ultimately also blocking xylem vessels [1,2,3,4,5,6,7,8].

Pathogenic *F. oxysporum* isolates notably cause disease on many important agronomical crops including grain and pasture legumes (chickpea, *Cicer arietinum*; field pea, *Pisum sativum*; lentil, *Lens culinaris*; lucerne/alfalfa, *Medicago sativa*), cotton (*Gossypium* species), banana (*Musa* species) and tomatoe (*Solanum lycopersicum*), and was ranked 5th out of the top 10 plant pathogens of scientific/economic importance [7,9,10,11]. The ability of specialised strains of *F. oxysporum* to cause disease on specific hosts is used to classify this pathogen into host-specific sub-species known as *formae speciales* (ff. spp.) [2,4,7] (singular *forma specialis*, abbreviated: f. sp.) and further into races depending on host cultivar resistance. For example, *F. oxysporum* f. sp. *lycopersici* infects tomato, f. sp. *conglutinans* infects canola and Brassica crops, and f. sp. *ciceris* infects chickpea (*Cicer arietinum*). *F. oxysporum* f. sp. *ciceris* is a major of pathogen of chickpea, the second most important legume crop worldwide [12], typically causing yield losses upwards of 10% with complete loss not uncommon under conducive conditions [8,13,14,15].

### 1.2. Host Jasmonate Signaling and F. oxysporum Disease Outcome

The *F. oxysporum*–host interaction is best understood in tomato and Arabidopsis, in the first instance due to identification of classical gene-for-gene mediated resistance and identification of *F. oxysporum* pathogenicity factors, and in the second instance to the use of in-depth genetic resources available to dissect host responses and the roles of hormone signalling [5,10,16,17,18,19]. In particular, evidence points towards contrasting roles for jasmonate (JA) signalling and JA-mediated defence in Arabidopsis resistance to *F. oxysporum* [10,18,20,21,22]. In Arabidopsis, JA-induced defences are critical for resistance against most fungal necrotrophs (e.g. *Botrytis cinerea, Alternaria brassicicola*) [23,24,25], but in the Arabidopsis–*F. oxysporum* interaction while JA-mediated defences contribute positively to *F. oxysporum* resistance, up-regulation of non-defensive components of JA-signalling such as senescence appear to promote susceptibility [17,18,26,27,28,29,30]. Together with JASMONATE ZIM DOMAIN (ZIM) proteins, the F-box protein CORONATINE INSENSITIVE 1 (COI1) forms part of the JA co-receptor complex for perception of the JA-signal (reviewed in [31]). Arabidopsis *coi1* mutants are insensitive to the JA-signal and remarkably in the absence of activated JA-mediated defences, *coi1* plants fail to develop disease symptoms following *F. oxysporum* infection [18,32,33]. This response appears dependent on the *formae speciales* used in disease assays, but consistent when used with those isolated off cabbage (*F. oxysporum* f. sp *conglutinans*) [16,18,26,32]. Interestingly, JA does not appear to play the same role in defence responses in tomato against *F. oxysporum* or other fungal necrotrophs and highlights the need to study host–pathogen interactions in model systems more closely related to the crop species in question [8,32,34,35]. In legumes, in addition to roles in pathogen interactions, JAs are also involved in regulating interactions with beneficial root-colonizing microorganisms [36,37,38,39,40,41].

JA is produced from the major plant plasma membrane lipid α-linolenic acid via the action of lipoxygenases and the octadecanoid biosynthetic pathway and is rapidly produced in response to pathogen or pest attack [31,42,43]. JA is then enzymatically converted into various derivatives such as JA–methyl ester (MeJA) and JA–amino acid conjugates, with JA-isoleucine the ligand for COI1-JAZ co-receptor recognition and activation of subsequent downstream JA-mediated responses [31,44,45,46,47]. Following pathogen or pest attack this may include the expression of defence-related genes such *PROTEINASE INHIBITORS* (*PIs*), *VEGETATIVE STORAGE PROTEINS* (*VSPs*), *CHITINASES* and *DEFENSINS.* Additionally, in a feedback loop, JA also induces the expression of genes that regulate its own biosynthesis such as *LIPOXYGENASES* (*LOX*), *OPDA REDUCTASE3* (*OPR3*) and *ALLENE OXIDE SYNTHASE* (*AOS*) [31,48,49,50].

### 1.3. Fusarium Wilt of Legumes

In legumes various sources of host resistance against *F. oxysporum* (f. sp. *ciceris*, chickpea; f. sp. *pisi*, pea; f. sp. *phaseoli*, bean; f. sp. *medicaginis*, *Medicago* species including alfalfa/lucerne) have been identified but the underlying genetic (e.g., *Resistance* genes) or molecular mechanisms are yet to be fully elucidated ([8,51,52,53,54,55] reviewed in [4]). To study the interaction between *F. oxysporum* and legume hosts we developed a model legume pathosystem utilising the model legume *Medicago truncatula* and its corresponding pathogenic f. sp. *medicaginis* isolated off alfalfa. While *M. truncatula* in its own right is an important pasture legume, it was also selected as a model species to study biological processes that are not easily undertaken in other legumes due to their large and/or complex genomes, and also to study processes unique to legumes (e.g., rhizobial symbioses) that cannot be studied in other model species such as Arabidopsis that do not undergo symbiotic interactions [36,56,57]. Indeed, it is reported Arabidopsis and *M. truncatula* lack extensive macrosynteny and share low levels of microsynteny (8%–10%) [57,58]. In addition to studying legume-specific biotic interactions, *M. truncatula* is also used as a model to dissect legume–pathogen interactions including necrotrophic fungal pathogens [38,39,59].

*M. truncatula* has been adopted by several groups worldwide as a model to identify and assess resistance mechanisms in legumes against *Fusarium* wilt [51,52,53] as well as other vascular wilt diseases and root rots such as *Verticillium* wilt (*Verticillium albo-atrum*) [60] and *Fusarium* root rot (*Fusarium solani*) [59]. In this review we describe current genomic and genetic resources available in *M. truncatula* and apply aspects to gain insight into host JA-responses during legume *F. oxysporum* interactions and how they may differ from JA-responses observed in Arabidopsis.

## 2. Genomic and Transcriptomic *M. truncatula* Resources

A reference *M. truncatula* genome was generated by The *M. truncatula* sequencing project in the A17 accession [61], with over 350 other lines from diverse genetic backgrounds now also under resequencing at greater than 5X coverage [62]. Expressed Sequence Tag (EST) resources and microarray platforms were also developed [57,63]. A gene expression “atlas” (*M. truncatula* Gene Expression Atlas, MtGEA [64]) was developed to display expression profiles of most *M. truncatula* genes covering major tissues (roots, nodules, stems, petioles, leaves, vegetative buds, flowers, seeds and seed pods), developmental time-series, and following various abiotic and biotic stresses [65,66] and more recently global gene expression data is available for viewing through the Genevestigator platform [67].

### Utilizing Gene Expression Resources to Study Medicago Responses to F. oxysporum Infection

To dissect JA-responses in the *M. truncatula–F. oxysporum* interaction we first aimed to identify *M. truncatula* genes responsive to JA. To do so, we inspected the *M. truncatula* Gene Expression Atlas for genes with the highest levels of up-regulated expression following MeJA treatment. This involved examining a dataset sourced from *M. truncatula* A17 cell suspensions treated with MeJA for 24 hours against a dataset from a control treatment for the same time period [68]. This process identified 245 genes with expression up-regulated ≥10-fold. Several *LOXs*, *PEPTIDASES*, *PI*s, *VSPs* and *CHITINASES* were amongst the most highly induced genes. As shown in Table 1, an assessment for biological processes enriched in the 245 gene set identified biological processes encompassing lipid and fatty acid biosynthetic/metabolic processes and response stimuli (biotic, chemical). Similar profiles are observed in Arabidopsis following MeJA [69] suggesting at least JA-regulated defence genes in *F.* *oxysporum* interactions identified in Arabidopsis may be transferrable to *M. truncatula*.

**Table 1:** Top 20 Gene Ontology (GO) biological process categories enriched in MeJA treated *Medicago* suspension cells. Based on significance of enrichment (False Discovery Rate (FDR) < 0.05) in genes expressed ≥10-fold in 24 h MeJA treated cell suspension relative to control treatment at the same time-point. Data sourced from MtGEA [64,65,66,68] and analyzed via agriGO [70].

Term	Description	FDR
GO:0044255	cellular lipid metabolic process	1.40E-17
GO:0019748	secondary metabolic process	1.40E-15
GO:0008610	lipid biosynthetic process	1.40E-15
GO:0032787	monocarboxylic acid metabolic process	2.40E-14
GO:0050896	response to stimulus	4.80E-14
GO:0009607	response to biotic stimulus	2.60E-13
GO:0010033	response to organic substance	2.30E-11
GO:0016053	organic acid biosynthetic process	3.10E-11
GO:0046394	carboxylic acid biosynthetic process	3.10E-11
GO:0006629	lipid metabolic process	3.80E-11
GO:0043436	oxoacid metabolic process	4.80E-11
GO:0006082	organic acid metabolic process	4.80E-11
GO:0019752	carboxylic acid metabolic process	4.80E-11
GO:0042180	cellular ketone metabolic process	4.80E-11
GO:0006720	isoprenoid metabolic process	5.20E-11
GO:0006631	fatty acid metabolic process	7.20E-11
GO:0006633	fatty acid biosynthetic process	1.90E-10
GO:0009719	response to endogenous stimulus	2.30E-10
GO:0042221	response to chemical stimulus	7.20E-10
GO:0008299	isoprenoid biosynthetic process	1.10E-09

**Figure 1:**
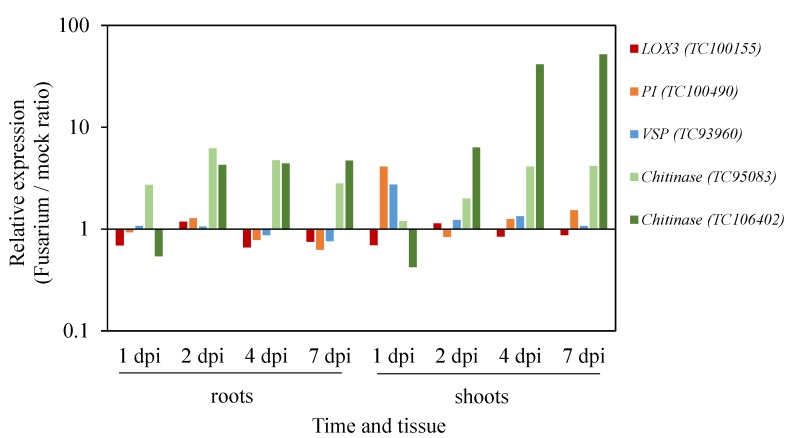
JA-inducible gene expression following *F. oxysporum* infection. *M. truncatula* A17 seedlings were inoculated with *F. oxysporum* (*Fom*-5190a) and root and shoot tissues harvested separately at 1, 2, 4 and 7 days post inoculation (dpi). Gene expression values were determined relative to the internal control *Beta-tubulin* gene for each mock or *Fusarium* treated sample. Values shown are fold-inductions in *Fusarium* treated samples relative to mock treated samples at the same time-point from the average of eight pooled plants.

**Figure 2:**
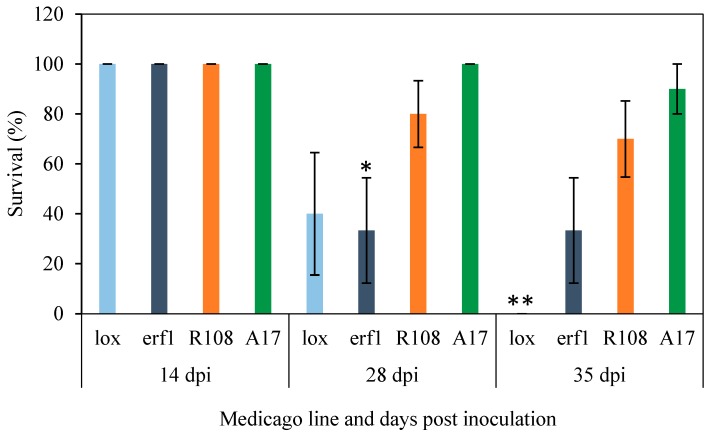
Susceptibility of JA-related *Tnt1*-insertion mutants to *F. oxysporum*. Seedlings were inoculated with *F. oxysporum* (*Fom*-5190a) and survival rates monitored over 35-days. Values are averages ± SE (n = 10). The *Tnt1*-insertion mutants are in the R108 background and their details noted in Table 2. A17 is included as a resistant control. Asterisks indicate values that are significantly different (** *p* < 0.01, * *p* < 0.05, Student’s t-test) from R108. Similar results were obtained in an independent experiment.

In Arabidopsis, following treatment with *F. oxysporum* a significant induction in JA-regulated defence gene expression is observed, where it is markedly greater in shoot (above ground) tissues than in roots [18,20,27]. To determine if similar patterns are observed in *M. truncatula* we treated *M. truncatula* A17 seedlings with *F. oxysporum* f. sp. *medicaginis* or a control (mock) treatment and examined whether candidate JA-responsive genes identified from the Gene Expression Atlas assessment and/or previously validated as MeJA-responsive [71], were induced in roots and shoots over an infection time-course (Figure 1). In this interaction, A17 displays moderate to strong resistance (Figure 2). Apart from one of the *CHITINASE* genes examined which showed a quicker and slightly greater induction in root tissues, in agreement with Arabidopsis the expression of JA-regulated defence genes tested (*PI, VSP, CHITINASES*) were predominantly up-regulated in shoot tissues. Ramírez-Suero and colleagues [53] also examined the expression of a *CHITINASE* and *PI* following *F. oxysporum* infection of *M. truncatula*, but only in root tissues. In that study they found A17 was susceptible to the isolate tested (*F. oxysporum* *f. sp. medicaginis* 179.29) and *CHITINASE* but not *PI* expression was up-regulated. A comparison between Arabidopsis and *M. truncatula* defensin-like (*DEFL*) genes, including those with JA-dependent expression, revealed differences in their gene expression patterns. Notably the majority of Arabidopsis *DEFL*s were expressed in inflorescences and not roots, while those in *M. truncatula* were predominantly expressed in root nodules [72].

We next examined expression of a candidate JA-biosynthesis *LOX* gene (*LOX3*) that is highly MeJA inducible [71] and of the *MtLOX* genes examined in MtGEA, exhibits the highest levels of expression. In Arabidopsis oxygenation of α-linolenic acid by one of its four 13-LOX proteins (AtLOX2, AtLOX3, AtLOX4, AtLOX6) is the first step in JA-biosynthesis with AtLOX2 the main contributor to JA production. Although the two remaining LOXs in Arabidopsis (9-LOXs AtLOX1 and AtLOX5) are not involved in JA-biosynthesis, they are involved in defence against bacterial pathogen attack (reviewed in [31]). In contrast to Arabidopsis where *F. oxysporum* infection induces the expression of *LOX* and other JA-biosynthesis genes (albeit to lower levels than JA-regulated defence genes) [18], we observed no induction in *M. truncatula* *LOX3* expression (Figure 1). This may suggest JA-signalling in *M. truncatula* differs to that in Arabidopsis in response to *F. oxysporum* infection. Further analysis of global JA-biosynthetic gene expression patterns and abundance of JA itself and its intermediates will be needed to unravel distinct JA-signalling processes in the *M. truncatula*-*F. oxysporum* interaction.

## 3. Genetic/Mutant Resources Available in *M. truncatula*

Several biological resources have been developed in *M. truncatula* to facilitate the elucidation of gene function [73,74,75]. This includes *M. truncatula* germplasm from diverse sources [62] as well as mutant populations generated through various techniques including Fast Neutron Bombardment [76], ethyl methane-sulfonate (EMS) [75,77,78] and *Tnt1* retrotransposon insertional mutagenesis [76]. The latter has become an excellent resource for reverse-genetics studies. Combining *Tnt1* mutant lines from the European Grain Legumes Integrated Project (GLIP) with the *M. truncatula* mutant database at the Noble Foundation [79,80], it is estimated the mutant resource contains insertions in ~90% of all genes. So far over 700,000 (high and low confidence) Flanking Sequence Tag (FST) information associated with insertions is available (accessed 08-10-2015).

### Utilizing M. truncatula Mutant Resources to Dissect Host JA-Responses under F. oxysporum Infection

In Arabidopsis the activation of JA-mediated defence responses promotes resistance to *F. oxysporum*, and the manipulation of transcriptional machinery that control these responses can alter disease outcome [81,82]. For example, mutation of the *MYC2* (JAI1/JIN1 JASMONATE-INSENSITIVE1) and *LBD20* (LATERAL ORGAN BOUNDARIES (LOB) DOMAIN-CONTAINING PROTEIN20) transcription factors (key JA-defence and MYC2-regulated transcription factors respectively), mutation of *PFT1* (PHYTOCHROME AND FLOWERING TIME1) and *ESR1* (KH-domain containing RNA-binding) both interacting components of the broader RNA polymerase II complex, or over-expression of the *ETHYLENE RESPONSE FACTORS ERF1* and *AtERF2* (transcriptional activators of JA-defences), results in increased resistance to *F. oxysporum* [17,20,21,29,30,83]. Interestingly however, overexpression of *MtERF1* in *M. truncatula* does not confer increased resistance to *F. oxysporum* [84] suggesting in combination with our examination of JA-mediated gene expression, unique differences in JA-responses exist between the two model systems.

To initiate a dissection of the role of host JA-responses in *M. truncatula*–*F. oxysporum* interactions we generated homozygous *Tnt1* mutants of *Mterf1* and a *LOX* (*Mtlox1*) obtained from the *M. truncatula* mutant database (Table 2). In Arabidopsis JA-biosynthesis itself does not appear to affect *F. oxysporum* disease outcome as mutants of the JA-biosynthesis pathway (e.g. *opr3, aos*) are as susceptible to *F. oxysporum* as wild-type plants and exogenous application of MeJA does not enhance resistance [18,19]. Studies in several plant species including *M. truncatula* have revealed roles for 9-LOXs in defence against fungal pathogens (reviewed in [85,86]). We therefore chose to assess a 9-LOX mutant instead of a 13-LOX. The *MtLOX* gene we selected here (*Medtr8g018430*) is annotated as encoding a 9S-LOX and when similarity against Arabidopsis proteins was conducted by Blastp to identify the Arabidopsis homologue, both LOX1 and LOX5 (9-LOXs) were the best hits. We infected the *Mtlox1* and *Mterf1* mutants alongside the reference genotype A17 and the accession R108 in which the mutant lines were generated. Both mutant lines showed a reduction in survival rate 28 days post infection (dpi) with all *Mtlox1* mutants dead by 35 dpi (Figure 2). Caution needs to be taken with the *Mtlox* mutant results as unlike the other genotypes assessed here which showed no reduction in survival following mock inoculation (water treatment), the *Mtlox1* seedlings were sensitive to the treatment process with a 60% survival rate at 35 dpi. The *Mterf1* mutant was also more susceptible to *F. oxysporum* infection as they succumb to disease pressure earlier than R108. Therefore while overexpression of *MtERF1* does not increase resistance to *F. oxysporum*, insertion inactivation appears to have an effect. In summary, our initial results suggest components of JA-signalling may be important determinants of disease outcome in *M. truncatula*.

**Table 2:** Details of *Tnt1*-insertion lines used in this study.

Putative Mutant	Medtr ID	TC	Insertion Line	Gene Function	Arabidopsis Homologue
*Mtlox1*	Medtr8g018430	TC132688	NF0217 insertion Ase8	9S-lipoxygenase	AT1G55020 *LOX1* AT3G22400 *LOX5*
*Mterf1*	Medtr4g100380	TC114237	NF1858 insertion 26	MtERF1-A transcription factor	AT4G17500 *ATERF-1*

Note, MtLOX1 shares the same percentage amino acid identity with both LOX1 and LOX5 from Arabidopsis. TC: tentative consensus.

## 4. Experimental Section

### 4.1. Plant Growth Conditions

Unless otherwise specified, all experiments were conducted with the *M. truncatula* accessions A17 or R108 grown under a short day light regime (8 h light:16 h dark) at 21 °C as described previously [71]. Seeds were germinated on damp filter paper, then transplanted into 30 mm Jiffy-7 peat pellets. Homozygous *M. truncatula Tnt1*-insertion lines were selected by PCR according to recommendations by [76].

### 4.2. Pathogen Assays

Plant pathogen assays were conducted using *F. oxysporum* f. sp. *medicaginis* strain *Fom*-5190a (BRIP 5190a/IMI 172838, collection number 19911) isolated from *Medicago sativa* by John. A. Irwin in Boonah (QLD, Australia) in 1973. *Fom*-5190a was maintained on sterile filter paper and grown on ½ strength potato dextrose agar. Three agar plugs were inoculated into flasks containing 100 mL of ½ strength potato dextrose broth and grown for 3 days at 28 °C/100 rpm. The resulting culture was drained through Miracloth (Calbiochem, San Diego, CA, USA,), spores pelleted through centrifugation, resuspended in sterile distilled water and the concentration adjusted to 1 × 10^6^ spores mL^−1^. For plant inoculations, two week old seedlings had roots protruding from the peat pellets trimmed then inoculated by placing the peat pellets in a petri dish of spore suspension for 5 min, followed by a further 1 mL of spore suspension added to the base of the hypocotyl. Inoculated pellets were transferred to growth trays lined with a plastic sheet and a thin layer of damp vermiculite, covered with a clear plastic dome to maintain humidity, and incubated under a long-daylight regime (16-h light/8-h dark) at 28 °C.

### 4.3. qRT-PCR

Quantitative-RT-PCR (qRT-PCR) experiments were performed on tissue collected after mock or *F. oxysporum* treatment. RNA extraction, cDNA synthesis and qRT-PCR were conducted as described by [21] using a CFX384 (Bio-Rad) system. Absolute gene expression levels relative to the validated reference gene *M. truncatula* *Beta-tubulin* were used for each cDNA sample using the equation: relative ratio gene of interest/*Beta-Tubulin* = (Egene^−Ct gene^)/(EBetatub^−Ct Betatub^) where Ct is the cycle threshold value. The gene specific primer sequences have been published previously [71] or are *Chitinase* TC95083 (F: 5’-ATGGCCAAACTCCAACTCTAAA-3’, R: 5’-ATTGAGGTGCTGCAGACAAGTA-3’) and *Chitinase* TC106402 (F: 5’-TTGCCGCGACTAGATCTTTTA-3’, R: 5’-GCGTCCATCTTCCAATAATCA-3’).

## 5. Conclusions

Based on the results presented herein, the detailed knowledgebase from JA-responses in Arabidopsis–*F. oxysporum* interactions are not all fully translatable to the interaction in *M. truncatula*. Legumes such as *M. truncatula* exist in complex interactions with both microbial pathogens and symbionts, thus the role of JA-signaling seems optimized to play different roles in response to the same pathogen of other hosts. It is envisaged the growing abundance of genomic, transcriptomic and genetic resources in *M. truncatula* will expedite the process of unravelling the roles of JA-signaling and responses controlling host disease outcome to devastating *Fusarium* wilt disease. Ultimately this knowledge will be disseminated to economically important legume crops used throughout the globe.
